# Bacterial Communities Associated With Healthy and Bleached Crustose Coralline Alga *Porolithon onkodes*

**DOI:** 10.3389/fmicb.2021.646143

**Published:** 2021-06-09

**Authors:** Fangfang Yang, Zhiliang Xiao, Zhangliang Wei, Lijuan Long

**Affiliations:** ^1^Key Laboratory of Tropical Marine Bio-resources and Ecology, South China Sea Institute of Oceanology, Chinese Academy of Sciences, Guangzhou, China; ^2^University of Chinese Academy of Sciences, Beijing, China

**Keywords:** bacterial community, coralline algae, metamorphosis, *Pocillopora damicornis*, *Porolithon onkodes*, settlement

## Abstract

Crustose coralline algae (CCA) play vital roles in producing and stabilizing reef structures and inducing the settlement and metamorphosis of invertebrate larvae in coral reef ecosystems. However, little is known about the bacterial communities associated with healthy and bleached CCA and their interactions with coral larval settlement. We collected samples of healthy, middle semi-bleached, and bleached CCA *Porolithon onkodes* from Sanya Bay in the South China Sea and investigated their influences on the larval settlement and metamorphosis of the reef-building coral *Pocillopora damicornis*. The larval settlement/metamorphosis rates all exceeded 70% when exposed to healthy, middle semi-bleached, and bleached algae. Furthermore, the compositions of bacterial community using amplicon pyrosequencing of the V3–V4 region of 16S rRNA were investigated. There were no obvious changes in bacterial community structure among healthy, middle semi-bleached, and bleached algae. *Alphaproteobacteria*, *Bacteroidetes*, and *Gammaproteobacteria* were dominant in all samples, which may contribute to coral larval settlement. However, the relative abundances of several bacterial communities varied among groups. The relative abundances of *Mesoflavibacter*, *Ruegeria*, *Nautella*, and *Alteromonas* in bleached samples were more than double those in the healthy samples, whereas *Fodinicurvata* and unclassified *Rhodobacteraceae* were significantly lower in the bleached samples. Additionally, others at the genus level increased significantly from 8.5% in the healthy samples to 22.93% in the bleached samples, which may be related to algal bleaching. These results revealed that the microbial community structure associated with *P. onkodes* generally displayed a degree of stability. Furthermore, bleached alga was still able to induce larval settlement and metamorphosis.

## Introduction

Crustose coralline algae (CCA) are considered as critical structural components of coral reef ecosystems. They play important roles in contributing to primary productivity, producing and stabilizing reef structures through CaCO_3_ deposition, and functioning as autogenic ecosystem engineers by the provision of three-dimensional habitat structure (Nelson, 2009; Tebben et al., 2015; van der Heijden and Kamenos, 2015). Furthermore, several CCA species have been shown to positively induce coral larval settlement and metamorphosis through a variety of mechanisms including chemical allelopathy and microbial induction (Morse and Hooker, 1988; Harrington et al., 2004; Gómez-Lemos et al., 2018). There are increasing evidences to suggest that algal–bacterial communities are species-specific and may play vital roles in larval settlement (Sneed et al., 2015; Yang et al., 2021), algal health, response to environmental stress, and defense against diseases (Harder et al., 2012; Singh and Reddy, 2014). However, algal–bacterial associations show highly dynamic relationships in response to environmental stress (Miranda et al., 2013; Quéré et al., 2019). For an improved understanding of the future health of coral reefs, it is important to determine the bacterial communities associated with CCA and how they shift in response to a disease or environmental stress.

Algal bleaching has recently occurred due to anthropogenic stressors and climate change, resulting in algal mortality (Martone et al., 2010; Cornwall et al., 2019). Previous studies have shown differences in bacterial communities between bleached and healthy organisms including corals and non-calcified macroalgae. The presence of some potential pathogens (i.e., *Vibrio shilonii*, *Nautella italica* R11, and *Phaeobacter gallaeciensis* LSS9) is related to bleaching disease (Kushmaro et al., 1996; Meron et al., 2011; Zozaya-Valdes et al., 2015; Rajasabapathy et al., 2020). To date, reports of microbial community variations between healthy and bleached CCA are very limited. Only two studies have investigated the microbiome associated with CCA-diseased tissue (*Neogoniolithon brassica-florida* and *N. mamillare*) (Meistertzheim et al., 2017; Quéré et al., 2019). Moreover, it is unclear whether changes in the bacterial communities associated with CCA affect the settlement of coral larvae.

*Porolithon onkodes* (Corallinales, Rhodophyta) is commonly found on tropical and subtropical coral reefs and is conspicuous on wave-resistant algal ridges (Dean et al., 2015). The species has been demonstrated to induce coral larval settlement and metamorphosis (Whitman et al., 2020). However, it is also one of the most vulnerable species to environmental change due to its highly soluble Mg-calcite skeleton (Ordoñez et al., 2019). Previous studies have revealed the physiological impacts of bleaching on *P. onkodes*, characterized by the loss of pink surface pigments, which occurs more frequently during summer months when temperatures and light radiation are elevated (Anthony et al., 2008; Bessell-Browne et al., 2017). It is unknown whether algal bleaching influences the larval settlement and distribution of coral populations.

*Pocillopora damicornis* is one of the most abundant and widespread hermaphrodite reef-building corals (Harriott, 1983) and is commonly used in experimental biology and physiology as a model species. There are many studies focusing on the microbiome of *P. damicornis*, which is mainly affiliated with γ-*Proteobacteria* (Bourne and Munn, 2005; Osman et al., 2020). However, in recent years, *P. damicornis* has suffered a degradation in reef ecosystems (Zheng et al., 2021). The coral recruitment and recovery depend mainly on the successful settlement and metamorphosis of coral larvae. A study has revealed that the larvae of *P. damicornis* preferentially settle on, or locate in close proximity to, a particular species of CCA (Yang et al., 2021). However, the mechanism of larval substrate choice and settlement specificity to CCA is unclear.

The main goal of the study was to investigate the bacterial communities associated with different health statuses of *P. onkodes* and their interactions with coral larval settlement. Our hypotheses are that (1) bacterial community associated with *P. onkodes* plays roles in larval settlement and metamorphosis and (2) bacterial community associated with *P. onkodes* is relatively stable but, however, their relative abundances differed. To test these hypotheses, in this study, healthy, middle semi-bleached, and bleached *P. onkodes* were collected from Sanya Bay, in the South China Sea. Their effects on coral larval survival, settlement, and bacterial community were investigated. The interactions between the bacterial community associated with *P. onkodes* and larval settlement, and potential pathogenic bacteria capable of causing algal bleaching, were analyzed.

## Materials and Methods

### Collection of CCA and Coral Larvae

Healthy, middle semi-bleached, and bleached *P. onkodes* were collected from Luhuitou fringing reef, Sanya Bay (18°12′N, 109°28′E), Hainan Island in the South China Sea in August, 2020. Fragments (3–5 cm) were collected from rocks using a hammer and chisel at 3–5-m depth. Each algal fragment was washed gently to remove epiphytes and then placed in an individual collecting bag in order to avoid contamination between specimens. Samples were then transported immediately to the laboratory. A total of nine samples, including three from healthy specimens (healthy group), three from the middle semi-bleached area between healthy and bleached specimens (middle group), and three from bleached specimens (bleached group), were immediately frozen by N_2_ and stored at −80°C for subsequent analysis of the bacterial community associated with *P. onkodes*. Other samples were cultured in flow-through tanks with filtered seawater at the Tropical Marine Biological Research Station in Sanya Bay for the settlement and metamorphosis assays. Ten colonies of coral *P. damicornis* were sampled at 2–3-m depth from Luhuitou fringing reef in August, 2020. Colonies were placed in flow-through buckets with filtered seawater. The released larvae were then collected in a chamber equipped with a 100-μm plankton mesh. The larvae were mixed and cultured with filtered seawater for the settlement and metamorphosis assays.

### Experimental Treatment and Larval Settlement Assays

To evaluate the larval settlement and metamorphosis responses to different health statuses of *P. onkodes*, four different treatments were conducted: control, healthy alga, middle alga, and bleached alga. Each experimental treatment had six replicates. Filtered seawater without the addition of alga was used as a negative control group. Fragments of *P. onkodes* were cut into 1 cm^2^ standardized surface area samples using a handheld grinding wheel. The effects of the following treated *P. onkodes* and extracts on larval settlement and metamorphosis were investigated: (i) live thalli extracted with either hot water (autoclave conditions, 121°C, 15 psi), cold water (27°C), ethanol, methanol, or methanol/chloroform (1:2, *v*/*v*). For all extractions, *P. onkodes* was extracted in 25 ml for 60 min; (ii) dried and autoclaved without water; and (iii) pink surface pigments were removed.

Ten *P. damicornis* larvae were randomly selected and added to individual wells of a six-well plate with 10 ml of 0.2 μm filtered seawater. The temperatures of the plates were maintained at 27°C by floating them in a seawater bath. The rates of larvae survival, metamorphosis, and settlement were calculated at 24 h with a dissecting microscope. The values were expressed as mean ± standard deviation. The following categories of larval behavior were observed in the assays: (i) dead larvae that had vanished or showed signs of degradation; (ii) swimming larvae with no response to cues; (iii) metamorphosis larvae that underwent morphological changes from a planula larva to a polyp, but without attachment to the substrate; and (iv) settlement and metamorphosis, defined as planulae firmly attached to the substratum and transforming into the coral primary polyp stage, respectively.

### DNA Extraction, PCR Amplification, and Sequencing of Microbial Communities

Bacterial communities of *P. onkodes* were investigated. Specially, microbial DNA was extracted from nine algal samples using the E.Z.N.A.^®^ Soil DNA Kit (Omega Bio-Tek, Norcross, GA, United States) according to manufacturer’s protocols. The final DNA concentration and purification were determined by a NanoDrop 2000 UV-Vis spectrophotometer (Thermo Fisher Scientific, Wilmington, DE, United States). DNA quality was checked by 1% agarose gel electrophoresis. The V3–V4 hypervariable regions of bacteria 16S rRNA gene were amplified with primers 338F (5′-ACTCCTACGGGAGGCAGCAG-3′) and 806R (5′-GGACTACHVGGGTWTCTAA T-3′) by a thermocycler polymerase chain reaction (PCR) system (GeneAmp 9700, ABI, Thermo Fisher Scientific, Wilmington, DE, United States) (Li et al., 2018; Yang et al., 2021). The PCR reactions were as follows: 3 min of denaturation at 95°C, 27 cycles of 30 s at 95°C, 30 s for annealing at 55°C, 45 s for elongation at 72°C, and a final extension for 10 min at 72°C. PCR reactions were performed in triplicate 20-μl mixtures containing 0.8 μl of each primer (5 μM), 4 μl of 5 × FastPfu Buffer, 2 μl of 2.5 mM dNTPs, 0.4 μl of FastPfu Polymerase, and 10 ng of template DNA. The PCR products were extracted from a 2% of agarose gel and purified using the AxyPrep DNA Gel Extraction Kit (Axygen Biosciences, Union City, CA, United States). They were then quantified using QuantiFluor^TM^ ST (Promega, Madison, WI, United States) according to the manufacturer’s protocols. Purified amplicons were pooled in equimolars and paired-end sequenced (2 × 300) on an Illumina MiSeq platform (Illumina, San Diego, CA, United States) according to the standard protocols by Majorbio Bio-Pharm Technology Co., Ltd. (Shanghai, China). The raw reads were deposited into the NCBI Sequence Read Archive under BioProject ID PRJNA685315 (accession numbers: SAMN17082550, SAMN17082551, SAMN17082552, SAMN17082553, SAMN17082554, SAMN17082555, SAMN17082556, SAMN17082557, and SAMN17082558).

Raw FASTQ files were demultiplexed, quality filtered by Trimmomatic, and merged by FLASH using the following criteria: (i) the reads were truncated at any site receiving an average quality score of 20 over a 50-bp sliding window; (ii) primers were exactly matched allowing two nucleotide mismatches, and reads containing ambiguous bases were removed; and (iii) sequences whose overlap exceeded 10 bp were merged according to their overlap sequence. Operational taxonomic units (OTUs) were clustered using UPARSE version 7.1^1^, and chimeric sequences were identified and removed using UCHIME (Osman et al., 2020). High-quality filtered tags with ≥97% similarity in nucleotide identity were clustered into same operational taxonomic units by OTU cluster analysis (Latif et al., 2020). The taxonomy of each 16S rRNA gene sequence was analyzed by the RDP Classifier algorithm^2^ against the Silva (SSU123) 16S rRNA database with a confidence threshold of 70%. Phylogenetic Investigation of Communities by Reconstruction of Unobserved States (PICRUSt2) software package was employed to predict the potential functional capabilities and differences among bacterial communities associated with different health statuses of *P. onkodes*.

### Statistical Analyses

The alpha diversity of bacterial community was analyzed using Shannon, Simpson’s, and Ace indices, based on the assigned OTUs (Latif et al., 2020; Osman et al., 2020). The beta diversity of bacterial community among different samples was assessed using a hierarchical cluster tree and principal coordinates analysis (PCoA) based on Bray–Curtis similarity. The relative abundances of bacterial phyla, class, and genera among the three algae groups were statistically analyzed using the Kruskal–Wallis *H*-test followed by the Scheffe’s *post hoc* test. Differences in the relative abundances of bacterial community composition between two groups were analyzed using the Welch’s *t*-test (White et al., 2009). The values in the settlement and metamorphosis assays were expressed as mean ± standard deviation. *P* < 0.05 was considered statistically significant, while *p* < 0.01 was considered extremely statistically significant.

## Results

### Responses of Coral Larvae to Healthy, Middle Semi-Bleached, and Bleached *P. onkodes*

**FIGURE 1:**
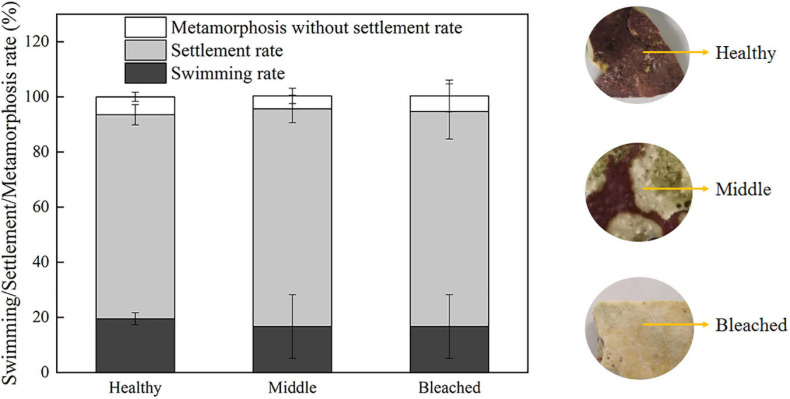
Settlement/metamorphosis rates of *P. damicornis* larvae exposed to different health statuses of *P. onkodes* at 24 h.

The effects of healthy, middle semi-bleached, and bleached *P. onkodes* on the settlement and metamorphosis of *P. damicornis* larvae were investigated. Larval survivorship in all groups was 100% within 24 h. As shown in Figure 1, the settlement rate was 76% at 24 h when exposed to healthy alga, which was similar to that in the middle and bleached algae at 78% (*p* = 0.32, Supplementary Table 1). Approximately 6% of coral larvae underwent morphological changes without attaching to the substratum when exposed to *P. onkodes* (Supplementary Figure 1). In the control group without cues, larvae were observed to actively swim throughout the exposure, and the settlement and metamorphosis rate was zero. These results suggested that bleached *P. onkodes* still induced larval settlement and metamorphosis.

**FIGURE 2:**
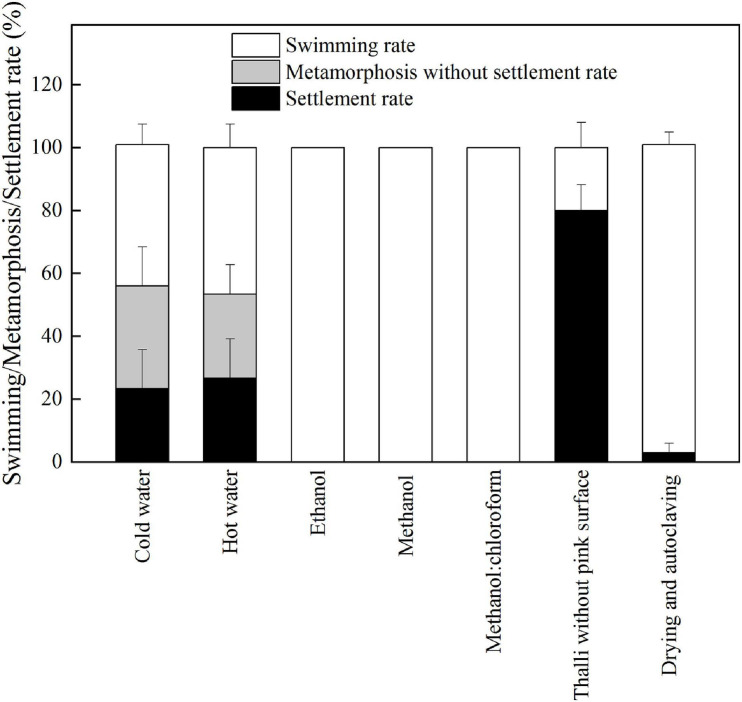
The responses of larval settlement and metamorphosis to the treated *P. onkodes* and algal extracts at 24 h.

As shown in Figure 2, *P. onkodes* lacking pink surface pigments induced high levels of settlement and metamorphosis (79%), which was consistent with healthy alga (*p* = 0.24). The result implied that the algal skeleton induced the settlement and metamorphosis of *P. damicornis* larvae rather than pink surface pigments. However, settlement rates decreased significantly when the larvae were exposed to algal extracts or autoclaved algae, especially those extracted with ethanol, methanol, and methanol/chloroform (0%). The settlement rates were 3, 23, and 26% for autoclaved algae, cold aqueous extracts, and hot aqueous extracts, respectively. Similarly, the metamorphosis rates decreased from 76% (healthy alga) to 53% (aqueous extracts). These results suggested that both the aqueous extracts and bacterial community associated with *P. onkodes* played important roles in larval settlement and metamorphosis.

### Bacterial Communities Associated With Different Health Statuses of *P. onkodes*

A total of 2,444 OTUs were predicted across all samples based on the 16S RNA gene database at the cut-off level of 97% (Supplementary Figure 2), among which 466 OTUs existed across all groups. The middle group (1,831 OTUs) had the highest number of OTUs, whereas the lowest number of OTUs was observed in the healthy group (848). Similarly, the middle group had the highest number of specific OTUs (828), followed by the bleached (372), and healthy groups (170).

**FIGURE 3:**
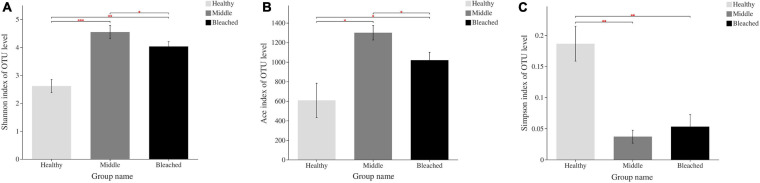
Richness and alpha diversity of bacterial communities associated with *P. onkodes* based on **(A)** Shannon index, **(B)** Ace index, and **(C)** Simpson index. ^∗^*p* ≤ 0.05, ^∗∗^*p* ≤ 0.01, and ^∗∗∗^*p* ≤ 0.001.

There were significant differences in the alpha diversity of bacterial community associated with different health statuses of *P. onkodes* according to the Shannon index (Figure 2). The diversity index of the middle group (4.54) was significantly higher than that of the healthy (4.03) and bleached groups (2.61) (Figure 3A, *p* = 0.001 and 0.042, respectively). Bacterial richness was calculated via the Ace index, which ranged from 607.5 to 1,299.5 (Figure 3B). Similarly, bacterial richness differed significantly between the healthy and bleached groups (*p* = 0.039). A significantly higher Ace index was observed in the bleached group compared with the healthy group. The Simpson’s index was significantly higher in the healthy group than in the middle and bleached groups (Figure 3C, *p* = 0.006 and 0.004, respectively). There was no significant difference in the Simpson’s index between the middle and bleached groups.

The beta diversity of bacterial community associated with different health statuses of *P. onkodes* was analyzed. Firstly, a hierarchical cluster tree of the bacterial community showed that the data were clustered in two distinct groups (Supplementary Figure 3). Group 1 contained the healthy samples, while group 2 included the middle and bleached samples. PCoA explained 67.54% of the observed variation and confirmed the output of the first method. The three samples within each group were relatively similar and clustered together. Healthy, middle, and bleached samples were grouped to the right, left, and left of the graph along PC1, respectively. The results of the two methods indicated that the middle and bleached groups had the highest level of similarity in bacterial community composition.

**FIGURE 4:**
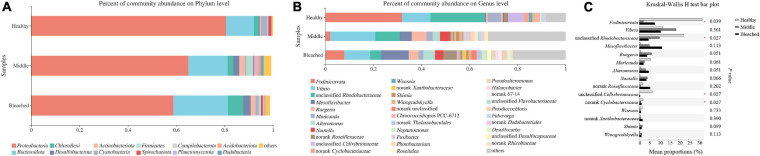
Microbial compositions at **(A)** the phylum level, **(B)** the genus level, and **(C)** the top 15 bacterial genus groups with significant differences among different health statuses of *P. onkodes*.

The bacterial community structure associated with different health statuses of *P. onkodes* was analyzed and presented in Figure 4. Overall, the structure of microbial communities was relatively stable among the three groups; however, the relative abundances of several bacteria differed. Specifically, the most dominant phylum was *Proteobacteria* with a relative abundance ranging from 59% in the bleached group and 81% in the healthy group (Figure 4A). Among *Proteobacteria*, *Alphaproteobacteria* was the predominant class, followed by *Gammaproteobacteria*. The average relative abundances of *Alphaproteobacteria* were 58, 33, and 32% in the healthy, middle, and bleached groups, respectively, while *Gammaproteobacteria* accounted for 22% (healthy group) to 31% (middle group) of the total relative abundance. *Bacteroidota*, mainly *Bacteroidetes*, was the second most dominant phylum with a relative abundance ranging from 12% in the healthy group and 22% in the bleached group. Other phyla with a relative abundance lower than 7%, including *Chloroflexi*, *Desulfobacterota*, *Actinobacteria*, *Cyanobacteria*, *Firmicutes*, and *Planctomycetes*, were also observed among all groups. As shown in Table 1, the Kruskal–Wallis *H*-test revealed that *Proteobacteria*, *Planctomycetota*, and *Acidobacteriota* exhibited statistically significant differences among the three groups. Additionally, the relative abundances of *Proteobacteria*, *Desulfobacterota*, *Actinobacteriota*, *Dadabacteria*, *Patescibacteria*, and *Campilobacterota* showed significant differences between the healthy and middle groups. For *Proteobacteria* and *Desulfobacterota*, there was a significant difference in their relative abundance between the healthy and bleached groups. Only the abundance of the phylum *Dadabacteria* differed significantly between the middle and bleached groups; however, the relative abundances were both lower than 2%.

**TABLE 1:** Statistical analysis of dominant bacterial phyla in bacterial communities associated with *P. onkodes*.

**Phylum**	***P* value**			
	**Among three groups**	**Healthy-middle**	**Healthy-bleached**	**Middle-bleached**
*Proteobacteria*	**0.039**	**0.022**	**0.013**	0.248
*Planctomycetota*	**0.027**	0.059	0.124	0.114
*Acidobacteriota*	**0.039**	0.165	0.212	0.663
*Desulfobacterota*	0.061	**0.015**	**0.035**	0.454
*Actinobacteriota*	0.061	**0.010**	0.196	0.663
*Dadabacteria*	0.051	**0.021**	0.228	**0.016**
*Campilobacterota*	0.061	**0.000**	0.117	0.389
*Patescibacteria*	0.061	**0.043**	0.368	0.863

*Bold numbers indicate p < 0.05.*

As shown in Figure 4B, *Fodinicurvata*, *Vibrio*, *Muricauda*, unclassified *Rhodobacteraceae*, and unclassified *Cellvibrionaceae* were the predominant genera in all groups. Among these, *Fodinicurvata* decreased from 31.9% in the healthy group to 2.1% in the middle group and 7.9% in the bleached group, whereas other genera increased from 8.5% in the healthy group to 32.5% in the middle group and 22.9% in the bleached group. Similarly, the relative abundances of unclassified *Rhodobacteraceae* were 22.6, 10.1, and 4.6% in the healthy, middle, and bleached groups, respectively. The abundance of unclassified *Cellvibrionaceae* ranged from 6.7% in the healthy group to 0.0% in the bleached group. *Muricauda* accounted for 5.7, 2.5, and 2.1% of the healthy, middle, and bleached groups, respectively. The relative abundances of *Mesoflavibacter* and other genera were higher in the bleached and middle groups compared with the healthy group.

**FIGURE 5:**
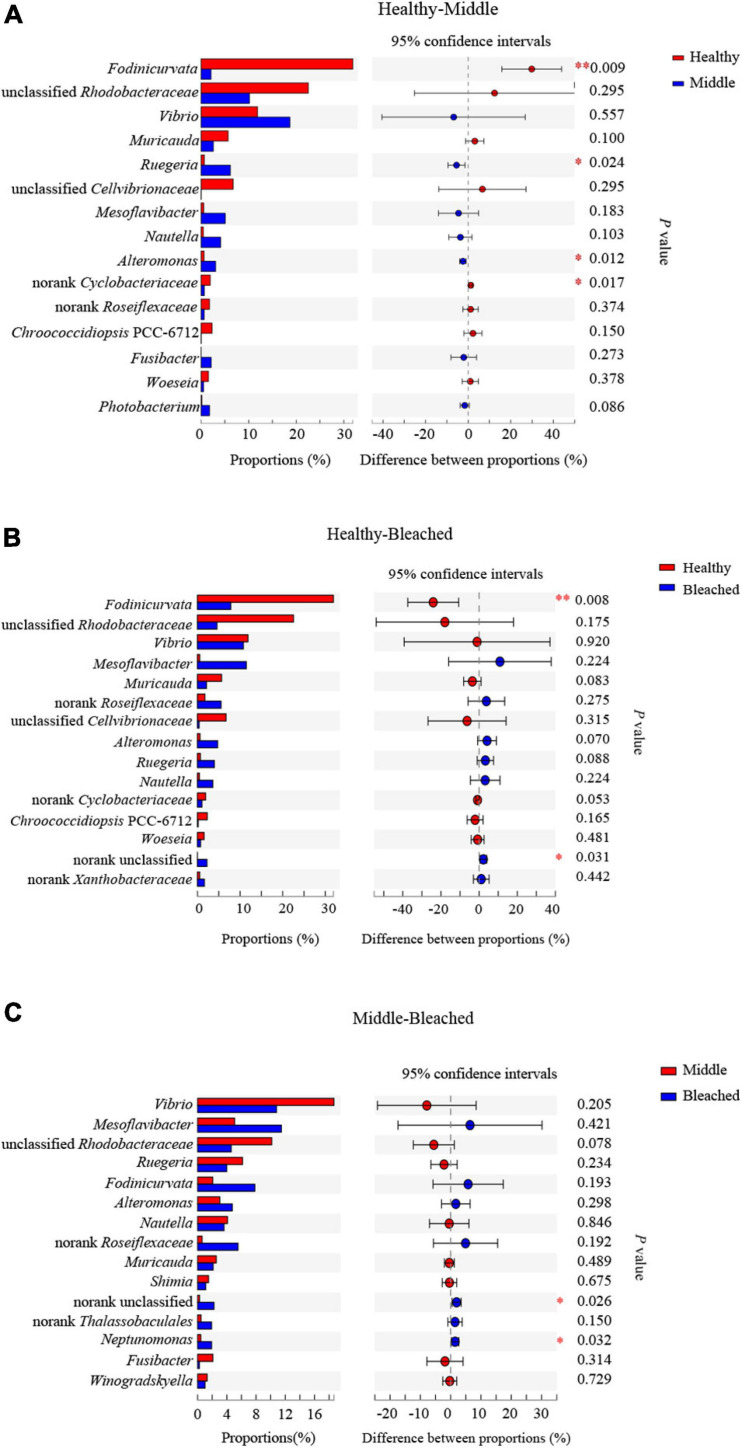
*Post hoc* testing of bacterial genus groups with significant differences **(A)** between healthy and middle groups, **(B)** between healthy and bleached groups, and **(C)** between middle and bleached groups. ^∗^*p* ≤ 0.05 and ^∗∗^*p* ≤ 0.01.

Statistical analysis revealed that the relative abundances of four bacterial genera were significantly different at the genus level among the groups, and these comprised *Fodinicurvata*, unclassified *Rhodobacteraceae*, unclassified *Cellvibrionaceae*, and norank *Xanthobacteraceae* (*p* < 0.05, Figure 4C). As shown in Figure 5, the relative abundance of *Fodinicurvata* was significantly higher in the healthy group compared with the other two groups; however, no significant difference was observed between the bleached and middle groups. The bleached alga had higher percentages of norank unclassified bacteria (*p* = 0.031), *Mesoflavibacter* (*p* = 0.224), *Ruegeria* (*p* = 0.088), *Nautella* (*p* = 0.224), and *Alteromonas* (*p* = 0.070) compared with healthy alga. Additionally, the relative abundances of *Ruegeria* and *Alteromonas* in the healthy group were significantly lower than those in the middle group (*p* = 0.024, 0.012, respectively).

**FIGURE 6:**
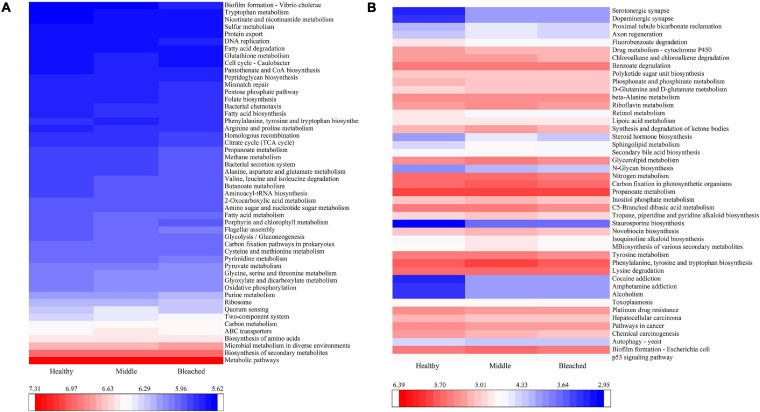
PICRUSt2 function prediction showing **(A)** the abundances of the top 50 of predicted pathways and **(B)** the abundances of the predicted pathways involved in diseases, metabolism, cellular processes, and organismal systems with significant differences among the three *P. onkodes* groups.

The PICRUSt2 program was used to predict metabolic functions in bacterial communities. At level 1, six pathways including metabolism, environmental information processing, genetic information processing, cellular processes, human diseases, and organismal systems were predicted. At level 2, the predictive pathways mainly focused on membrane transport, signal transduction, translation, carbohydrate metabolism, and infectious disease. Figure 6A reveals major predictive pathways at level 3. The biosynthesis of amino acids, ABC transporters, two-component system, carbon metabolism, and quorum sensing was observed in all groups. Among these pathways, quorum sensing, two-component system, bacterial secretion system, and bacterial chemotaxis may be related to larval settlement. As shown in Figure 6B, there were no significant differences in these pathways among groups (*p* > 0.05), which may be the main reason for the lack of change in settlement rates when coral larvae were exposed to different health statuses of *P. onkodes*. However, the function of several bacterial flora involved in disease and metabolism changed significantly among different groups (Figure 6B). Specifically, the expression of alcoholism, amphetamine addiction, and cocaine addiction in disease pathway; staurosporine biosynthesis and glycan biosynthesis in metabolism pathway; dopaminergic synapse and serotonergic synapse in organismal systems increased significantly in the middle and bleached groups compared with the healthy group (*p* < 0.05). Conversely, the abundances of retinol metabolism, phosphonate and phosphinate metabolism, chloroalkane and chloroalkene degradation, and fluorobenzoate degradation in metabolism pathway were lower in the middle and bleached groups than those in the healthy group (*p* < 0.05).

## Discussion

### Stability of Bacterial Communities Associated With Different Health Statuses of *P. onkodes*

Crustose coralline algae are the most abundant and important calcified macroalgae worldwide (Nelson, 2009). It has been demonstrated that each CCA harbors a unique bacterial community (Cavalcanti et al., 2014; Sneed et al., 2015; Brodie et al., 2016). Hollants et al. (2013) reviewed 161 macroalgal–bacterial studies and reported that *Gammaproteobacteria*, *Alphaproteobacteria*, *Bacteroidetes*, *Firmicutes*, and *Actinobacteria* represented the core microbiome of these macroalgae. Sneed et al. (2015) showed that the microbiomes of four CCA species were similarly dominated by *Gammaproteobacteria*, *Alphaproteobacteria*, and *Actinobacteria* but that *Bacteroidetes* was not the most dominant phylum recorded. Different results were observed in *Corallina officinalis*, which had high abundances of *Gammaproteobacteria*, *Alphaproteobacteria*, *Bacteroidetes*, and *Flavobacteria* and a low proportion of *Firmicutes* (Brodie et al., 2016). In the present study, the bacterial communities associated with different health statuses of *P. onkodes* (i.e., healthy, middle, and bleached) were determined. We found that bacterial community compositions were similar among groups. *Alphaproteobacteria*, *Gammaproteobacteria*, and *Bacteroidetes* comprised the core bacterial microbiome members of *P. onkodes*; their relative abundance accounted for 80–90%. Additionally, *P. onkodes* had a relatively low proportion of *Actinobacteria* and lacked *Firmicutes* in its core microbiome, compared with previously reported algal species (Hollants et al., 2013). These results imply that the overall bacterial community composition associated with *P. onkodes* is relatively conserved.

The relative abundances of bacterial communities associated with algae are affected by sea water temperatures, pH, habitats, and disease (Webster et al., 2011; Miranda et al., 2013; Meistertzheim et al., 2017). Brodie et al. (2016) reported that the OTU number, Chao1 richness, and Shannon diversity index of the *C. officinalis* microbiome were significantly affected by different intertidal habitats. Greater abiotic stress experienced in the upper intertidal could enhance the overall richness and diversity in the bacterial community. Our study revealed that diversity and richness of the bacterial community based on the abundance of OTUs were correlated with algal health statuses, which increased when the alga was bleached. Additionally, there were differences in the relative abundances of several bacteria among different groups. For example, the bacterial sequences that were classified as other genera significantly increased in the bleached group, indicating that unknown genera or new pathogens increased when the *P. onkodes* was bleached. These findings suggested that the algal health status affected the relative abundance of several microbial species but did not fundamentally impact the bacterial community structure. This is consistent with the findings of Hadaidi et al. (2017), who found that coral health condition had no significant effect on bacterial community composition but detected a significant difference in abundance.

### Roles of *P. onkodes*-Associated Bacterial Communities in Coral Larval Settlement and Metamorphosis

Crustose coralline algae have been shown to facilitate the settlement and metamorphosis of coral larvae (such as *Pocillopora* and *Acropora*) (Tebben et al., 2011, 2015). However, their capacities to induce coral larval settlement and metamorphosis differ among phylogenetically distinct CCA species. For example, *P. onkodes* (formerly *Hydrolithon onkodes*) and *Porolithon gardineri* induce high levels of larval settlement, whereas *Neogoniolithon fosliei* induces a lower settlement rate at 4.7%. A potential reason for these species-specific differences is that each CCA species might harbor distinct bacterial communities or algal components that affect coral larval settlement and metamorphosis (Johnson et al., 1991; Tebben et al., 2015; Gómez-Lemos et al., 2018; Quinlan et al., 2019). Tebben et al. (2015) found that chemical cues (i.e., glycoglycerolipids and polysaccharides) derived from CCA could trigger larval settlement and metamorphosis. Recent studies have suggested that the bacterial communities associated with CCA play important roles in larval settlement (Sneed et al., 2015; Siboni et al., 2020). Coral larvae may selectively settle on CCA species through the recognition of bacterial communities on CCA (Sneed et al., 2015). To the best of our knowledge, the present study is the first to investigate the larval settlement response to different health statuses of CCA (i.e., healthy, middle, and bleached) and their bacterial community composition. Interestingly, we found that bleached *P. onkodes* could still induce the settlement and metamorphosis of coral larvae. Furthermore, the aqueous extracts of *P. onkodes* could induce larval settlement and metamorphosis; however, the settlement and metamorphosis rates were lower than those associated with healthy alga. This finding indicated that the algal extracts and CCA-associated bacterial communities play vital roles in larval settlement and metamorphosis. This raises the question of which bacteria are associated with the induction. As previously mentioned, the bacterial community composition was similar among the three groups. Furthermore, there were no significant differences in quorum sensing, two-component system, bacterial secretion system, and bacterial chemotaxis pathways among the groups in the PICRUSt2 analysis. Therefore, it is speculated that bacteria involved in these pathways may be related to the larval settlement, which is one of the potential reasons why all three groups significantly induced *P. damicornis* larval settlement.

### Importance of Bacterial Communities Associated With *P. onkodes* for Algal Health

Marine bacteria have important functions for the health, performance, and resilience of multicellular organisms, as demonstrated for epiphytic bacteria associated with macroalgae (Harder et al., 2012; Egan et al., 2013; Singh and Reddy, 2014). However, their negative influences are also increasingly recognized in the disease of organisms. When homeostasis in organism-associated bacterial community is disrupted, the organism health can be affected (de Castro et al., 2010; Pollock et al., 2019). In the present study, the abundances of predicted pathways involved in infectious diseases, cell growth and death, immune system, and metabolism of other amino acids significantly changed in the diseased alga compared with healthy alga. This indicates that algal diseases affect the immune system, metabolism, growth, and death. Regarding the question of which bacteria are associated with disease, previous studies have shown that coral or macroalgal diseases are caused by multiple bacteria (Largo et al., 1995; Joyner et al., 2015). Among these bacteria, some genera of *Rhodobacteraceae* and *Rhizobiaceae*, the most dominant families in algae and coral (Wang et al., 2020), have been thought to be highly related to stony coral disease (Cárdenas et al., 2012; Meyer et al., 2019; Rosales et al., 2020). Our study revealed that the relative abundances of *Rhodobacteraceae* and *Rhizobiaceae* differed between the bleached and healthy groups. Therefore, the abundances of *Rhodobacteraceae* and *Rhizobiaceae* may be correlated with algal disease.

Bleaching disease, which is characterized by localized pigment loss, is considered to be a primary algal disease (Campbell et al., 2014). The bleaching disease of *Delisea pulchra* is one of the best-studied models (Fernandes et al., 2012; Cooper and Smith, 2015). Kumar et al. (2016) found that *Alteromonas* sp. belonging to the family *Alteromonadaceae* (*Gammaproteobacteria*) could be an opportunistic pathogen that causes the bleaching of *D. pulchra*, although healthy individuals also had a low abundance of *Alteromonas* sp. A similar result was observed in the present study whereby bleached *P. onkodes* had a higher abundance of *Alteromonas* sp. Additionally, *Nautella* and *Phaeobacter* were also considered as pathogens that cause symptomatic bleaching in algal sporelings during *in vitro* infection assays (Case et al., 2011; Fernandes et al., 2011; Campbell et al., 2014). In the current study, *Nautella* and *Phaeobacter* were not found in the healthy group, but *Nautella* was present in the semi-bleached and bleached groups. Therefore, it is speculated that *Nautella* and *Alteromonas* may be potential pathogens capable of causing algal bleaching. Further investigation into multiple pathogens resulting in algal bleaching is warranted.

In conclusion, relatively stable bacterial communities were observed in bleached, middle semi-bleached, and healthy *P. onkodes*. *Alphaproteobacteria*, *Gammaproteobacteria*, and *Bacteroidetes* were the dominant phyla in all algal samples, although there were apparent differences in the relative abundance of bacterial phyla. These abundant and ubiquitous bacterial taxa were identified as core bacterial microbiome members of *P. onkodes*. Furthermore, and noteworthy, the bleaching of *P. onkodes* did not affect the coral larval settlement of *P. damicornis*, which was likely related to its conserved bacterial communities. Additionally, there was a lower relative abundance of *Fodinicurvata* and higher relative abundances of *Mesoflavibacter*, *Ruegeria*, *Nautella*, and *Alteromonas* in the bleached alga compared with healthy alga. Therefore, *Nautella* and *Alteromonas* may be potential pathogens that result in algal bleaching.

## Data Availability Statement

The datasets presented in this study can be found in online repositories. The names of the repository/repositories and accession number(s) can be found below: https://www.ncbi.nlm.nih.gov/, BioProject ID PRJNA685315.

## Author Contributions

FY designed and performed the experiments, analyzed the data, and wrote the manuscript. ZX and ZW participated in larval settlement assays. LL conceived the experiments and revised the manuscript. All authors contributed to manuscript writing and provided final approval for publication.

## Conflict of Interest

The authors declare that the research was conducted in the absence of any commercial or financial relationships that could be construed as a potential conflict of interest.

## Footnotes

1 http://drive5.com/uparse/

2 http://rdp.cme.msu.edu/

## Supplementary Material

Supplementary Figure 1 Coral larvae exposed to **(A)** healthy, **(B)** middle, and **(C)** bleached *P. onkodes*. Red arrows represent different health statuses of *P. onkodes*, while green arrows indicate coral larvae.

Supplementary Figure 2 Venn diagram showing the number of OTUs in healthy, middle, and bleached *P. onkodes* based on the 16S RNA gene database at the cut-off level of 97%.

Supplementary Figure 3 The beta diversity of bacterial communities associated with *P. onkodes* based on a hierarchical cluster tree and principal coordinates analysis (PCoA). The values of axes 1 and 2 represent the percentages that can be explained by the corresponding axis.

Supplementary Table 1 Analysis of variance on larval settlement/metamorphosis/swimming rates exposed to different health statuses of *P. onkodes*.

## References

[B1] Anthony K. R. N. Kline D. I. Diaz-Pulido G. Dove S. Hoegh-Guldberg O. (2008). Ocean acidification causes bleaching and productivity loss in coral reef builders. *PNAS* 105 17442–17446. 10.1073/pnas.0804478105 18988740 PMC2580748

[B2] Bessell-Browne P. Negri A. P. Fisher R. Clode P. L. Jones R. (2017). Impacts of light limitation on corals and crustose coralline algae. *Sci. Rep.* 7:11553. 10.1038/s41598-017-11783-z 28912462 PMC5599546

[B3] Bourne D. G. Munn C. B. (2005). Diversity of bacteria associated with the coral Pocillopora damicornis from the Great Barrier Reef. *Environ. Microbiol.* 7 1162–1174. 16011753 10.1111/j.1462-2920.2005.00793.x

[B4] Brodie J. Williamson C. Barker G. L. Walker R. H. Briscoe A. Yallop M. (2016). Characterising the microbiome of Corallina officinalis, a dominant calcified intertidal red alga. *FEMS Microbiol. Ecol.* 92:fiw110. 27222222 10.1093/femsec/fiw110 PMC5831014

[B5] Campbell A. H. Vergés A. Steinberg P. D. (2014). Demographic consequences of disease in a habitat-forming seaweed and impacts on interactions between natural enemies. *Ecology* 95 142–152. 10.1890/13-0213.1 24649654

[B6] Cárdenas A. Rodriguez-R L. M. Pizarro V. Cadavid L. F. Arévalo-Ferro C. (2012). Shifts in bacterial communities of two Caribbean reef-building coral species affected by white plague disease. *ISME J.* 6 502–512. 10.1038/ismej.2011.123 21955993 PMC3280130

[B7] Case R. J. Longford S. R. Campbell A. H. Low A. Tujula N. Steinberg P. D. (2011). Temperature induced bacterial virulence and bleaching disease in a chemically defended marine macroalga. *Environ. Microbiol.* 13 529–537. 10.1111/j.1462-2920.2010.02356.x 20946533

[B8] Cavalcanti G. S. Gregoracci G. B. Santos E. O. D. Silveira C. B. Meirelles P. M. Longo L. L. (2014). Physiologic and metagenomic attributes of the rhodoliths forming the largest CaCO3 bed in the South Atlantic Ocean. *ISME J.* 8 52–62. 10.1038/ismej.2013.133 23985749 PMC3869012

[B9] Cooper M. B. Smith A. G. (2015). Exploring mutualistic interactions between microalgae and bacteria in the omics age. *Curr. Opin. Plant. Biol.* 26 147–153. 10.1016/j.pbi.2015.07.003 26318329

[B10] Cornwall C. E. Diaz-Pulido G. Comeau S. (2019). Impacts of ocean warming on coralline algal calcification: meta-analysis, knowledge gaps, and key recommendations for future research. *Front. Mar. Sci.* 6:186. 10.3389/fmars.2019.00186

[B11] de Castro A. P. Araújo S. D. Reis A. M. M. Moura R. L. Francini-Filho R. B. Pappas G. Jr. (2010). Bacterial community associated with healthy and diseased reef coral Mussismilia hispida from Eastern Brazil. *Microb. Ecol.* 59 658–667. 10.1007/s00248-010-9646-1 20352207

[B12] Dean A. J. Steneck R. S. Tager D. Pandolfi J. M. (2015). Distribution, abundance and diversity of crustose coralline algae on the Great Barrier Reef. *Coral Reefs.* 34 581–594. 10.1007/s00338-015-1263-5

[B13] Egan S. Harder T. Burke C. Steinberg P. Kjelleberg S. Thomas T. (2013). The seaweed holobiont: understanding seaweed–bacteria interactions. *FEMS Microbiol. Rev.* 37 462–476. 10.1111/1574-6976.12011 23157386

[B14] Fernandes N. Case R. J. Longford S. R. Seyedsayamdost M. R. Steinberg P. D. Kjelleberg S. (2011). Genomes and virulence factors of novel bacterial pathogens causing bleaching disease in the marine red alga Delisea pulchra. *PLoS One* 6:e27387. 10.1371/journal.pone.0027387 22162749 PMC3230580

[B15] Fernandes N. Steinberg P. Rusch D. Kjelleberg S. Thomas T. (2012). Community structure and functional gene profile of bacteria on healthy and diseased thalli of the red seaweed Deliseapulchra. *PLoS One* 7:e50854. 10.1371/journal.pone.0050854 23226544 PMC3513314

[B16] Gómez-Lemos L. A. Doropoulos C. Bayraktarov E. Diaz-Pulido G. (2018). Coralline algal metabolites induce settlement and mediate the inductive effect of epiphytic microbes on coral larvae. *Sci. Rep.* 8:17557. 30510183 10.1038/s41598-018-35206-9 PMC6277392

[B17] Hadaidi G. Röthig T. Yum L. K. Ziegler M. Arif C. Roder C. (2017). Stable mucus-associated bacterial communities in bleached and healthy corals of Porites lobate from the Arabian Seas. *Sci. Rep.* 7:45362. 10.1038/srep45362 28361923 PMC5374439

[B18] Harder T. Campbell A. H. Egan S. Steinberg P. D. (2012). Chemical mediation of ternary interactions between marine holobionts and their environment as exemplified by the red alga Delisea pulchra. *J. Chem. Ecol.* 38 442–450. 10.1007/s10886-012-0119-5 22527059

[B19] Harrington L. Fabricius K. De’ath G. Negri A. (2004). Recognition and selection of settlement substrata determine post-settlement survival in corals. *Ecology* 85 3428–3437. 10.1890/04-0298

[B20] Harriott V. J. (1983). Reproductive seasonality, settlement, and post-settlement mortality of *Pocillopora damicornis* (Linnaeus), at Lizard Island, Great Barrier Reef. *Coral Reefs.* 2 151–157. 10.1007/BF00336721

[B21] Hollants J. Leliaert F. De Clerck O. Willems A. (2013). What we can learn from sushi: a review on seaweed-bacterial associations. *FEMS Microbiol. Ecol.* 83 1–16. 10.1111/j.1574-6941.2012.01446.x 22775757

[B22] Johnson C. R. Sutton D. Olson R. Giddins R. (1991). Settlement of crown-of-thorns starfish: role of bacteria on surfaces of coralline algae and a hypothesis for deepwater recruitment. *Mar. Ecol. Prog. Ser.* 71 143–162.

[B23] Joyner J. L. Sutherland K. P. Kemp D. W. Berry B. Griffin A. Porter J. W. (2015). Systematic analysis of white pox disease in Acropora palmata of the Florida Keys and role of *Serratia marcescens*. *Appl. Environ. Microbiol.* 81 4451–4457. 10.1128/AEM.00116-15 25911491 PMC4475871

[B24] Kumar V. Zozaya-Valdes E. Kjelleberg S. Thomas T. Egan S. (2016). Multiple opportunistic pathogens can cause a bleaching disease in the red seaweed Delisea pulchra. *Environ. Microbiol.* 18 3962–3975. 27337296 10.1111/1462-2920.13403

[B25] Kushmaro A. Loya Y. Fine M. Rosenberg E. (1996). Bacterial infection and coral bleaching. *Nature* 380 396–396. 10.1038/380396a0

[B26] Largo D. B. Fukami K. Nishijima T. (1995). Occasional pathogenic bacteria promoting ice-ice disease in the carrageenan-producing red algae Kappaphycus alvarezii and Eucheuma denticulatum (Solieriaceae, Gigartinales, Rhodophyta). *J. Appl. Phycol.* 7 545–554. 10.1007/BF00003941

[B27] Latif S. Bibi S. Kouser R. Fatimah H. Farooq S. Naseer S. (2020). Characterization of bacterial community structure in the rhizosphere of Triticum aestivum L. *Genomics* 112 4760–4768. 10.1016/j.ygeno.2020.07.031 32712294

[B28] Li Y. F. Yang N. Liang X. Yoshida A. Osatomi K. Power D. (2018). Elevated seawater temperatures decrease microbial diversity in the gut of Mytilus coruscus. *Front. Physiol.* 10:839. 10.3389/fphys.2018.00839 30042689 PMC6049046

[B29] Martone P. T. Alyono M. Stites S. (2010). Bleaching of an intertidal coralline alga: untangling the effects of light, temperature, and desiccation. *Mar. Ecol. Prog. Ser.* 416 57–67. 10.3354/meps08782

[B30] Meistertzheim A. L. Nugues M. M. Quéré G. Galand P. E. (2017). Pathobiomes differ between two diseases affecting reef building coralline algae. *Front. Microbiol.* 8:1686. 10.3389/fmicb.2017.01686 28919890 PMC5585562

[B31] Meron D. Atias E. Kruh L. I. Elifantz H. Minz D. Fine M. (2011). The impact of reduced pH on the microbial community of the coral Acropora eurystoma. *ISME J.* 5 51–60. 10.1038/ismej.2010.102 20668489 PMC3105665

[B32] Meyer J. L. Castellanos-Gell J. Aeby G. S. Häse C. C. Ushijima B. Paul V. J. (2019). Microbial community shifts associated with the ongoing stony coral tissue loss disease outbreak on the florida reef tract. *Front. Microbiol.* 10:2244. 10.3389/fmicb.2019.02244 31608047 PMC6769089

[B33] Miranda L. N. Hutchison K. Grossman A. T. Brawley S. H. (2013). Diversity and abundance of the bacterial community of the red macroalga Porphyra umbilicalis: did bacterial farmers produce macroalgae? *PLoS One* 8:e58269. 10.1371/journal.pone.0058269 23526971 PMC3603978

[B34] Morse D. E. Hooker N. (1988). Control of larval metamorphosis and recruitment in sympatric agariciid corals. *J. Exp. Mar. Biol. Ecol.* 116 193–217. 10.1016/0022-0981(88)90027-5

[B35] Nelson W. (2009). Calcified macroalgae–critical to coastal ecosystems and vulnerable to change: a review. *Mar. Freshw. Res.* 60 787–801.

[B36] Ordoñez A. Wangpraseurt D. Lyndby N. H. Kühl M. Diaz-Pulido G. (2019). Elevated CO2 leads to enhanced photosynthesis but decreased growth in early life stages of reef building coralline algae. *Front. Mar. Sci.* 5:495. 10.3389/fmars.2018.00495

[B37] Osman E. O. Suggett D. J. Voolstra C. R. Pettay D. T. Clark D. R. Pogoreutz C. (2020). Coral microbiome composition along the northern Red Sea suggests high plasticity of bacterial and specificity of endosymbiotic dinoflagellate communities. *Microbiome* 8:8. 10.1186/s40168-019-0776-5 32008576 PMC6996193

[B38] Pollock F. J. Lamb J. B. van de Water J. A. J. M. Smith H. A. Schaffelke B. Willis B. L. (2019). Reduced diversity and stability of coral-associated bacterial communities and suppressed immune function precedes disease onset in corals. *R. Soc. Open Sci.* 6:190355. 10.1098/rsos.190355 31312497 PMC6599770

[B39] Quéré G. Intertaglia L. Payri C. Galand P. E. (2019). Disease specific bacterial communities in a coralline algae of the Northwestern Mediterranean Sea: a combined culture dependent and-Independent approach. *Front. Microbiol.* 10:1850. 10.3389/fmicb.2019.01850 31555220 PMC6722220

[B40] Quinlan Z. A. Ritson-Williams R. Carroll B. J. Carlson C. A. Nelson C. E. (2019). Species-specific differences in the microbiomes and organic exudates of crustose coralline algae influence bacterioplankton communities. *Front. Microbiol.* 10:2397. 10.3389/fmicb.2019.02397 31781048 PMC6857149

[B41] Rajasabapathy R. Ramasamy K. P. Manikandan B. Mohandass C. James R. A. (2020). Bacterial communities associated with healthy and diseased (skeletal growth anomaly) reef coral Acropora cytherea from Palk Bay, India. *Front. Mar. Sci.* 7:92. 10.3389/fmars.2020.00092

[B42] Rosales S. M. Clark A. S. Huebner L. K. Ruzicka R. R. Muller E. M. (2020). *Rhodobacterales* and *Rhizobiales* are associated with stony coral tissue loss disease and its suspected sources of transmission. *Front. Microbiol* 11:681. 10.3389/fmicb.2020.00681 32425901 PMC7212369

[B43] Siboni N. Abrego D. Puill-Stephan E. King W. L. Bourne D. G. Raina J. B. (2020). Crustose coralline algae that promote coral larval settlement harbor distinct surface bacterial communities. *Coral Reefs.* 39 1703–1713. 10.1007/s00338-020-01997-5

[B44] Singh R. P. Reddy C. R. K. (2014). Seaweed-microbial interactions: key function of seaweed-associated bacteria. *FEMS Microb. Ecol.* 88 213–230. 10.1111/1574-6941.12297 24512602

[B45] Sneed J. M. Ritson-Williams R. Paul V. J. (2015). Crustose coralline algal species host distinct bacterial assemblages on their surfaces. *ISME J.* 9 2527–2536. 10.1038/ismej.2015.67 25918832 PMC4611515

[B46] Tebben J. Motti C. A. Siboni N. Tapiolas D. M. Negri A. P. Schupp P. J. (2015). Chemical mediation of coral larval settlement by crustose coralline algae. *Sci. Rep.* 5:10803. 10.1038/srep10803 26042834 PMC4650656

[B47] Tebben J. Tapiolas D. M. Motti C. A. Abrego D. Negri A. P. Blackall L. L. (2011). Induction of larval metamorphosis of the coral Acropora millepora by tetrabromopyrrole isolated from a Pseudoalteromonas bacterium. *PLoS One* 6:e19082. 10.1371/journal.pone.0019082 21559509 PMC3084748

[B48] van der Heijden L. H. Kamenos N. A. (2015). Reviews and syntheses: calculating the global contribution of coralline algae to total carbon burial. *Biogeosciences* 12 6429–6441. 10.5194/bg-12-6429-2015

[B49] Wang J. Lu J. Zhang Y. Wu J. (2020). Microbial ecology might serve as new indicator for the influence of green tide on the coastal water quality: assessment the bioturbation of Ulva prolifera outbreak on bacterial community in coastal waters. *Ecol. Indic.* 113:106211. 10.1016/j.ecolind.2020.106211

[B50] Webster N. S. Soo R. Cobb R. Negri A. P. (2011). Elevated seawater temperature causes a microbial shift on crustose coralline algae with implications for the recruitment of coral larvae. *ISME J.* 5 759–770. 10.1038/ismej.2010.152 20944682 PMC3105747

[B51] White J. R. Nagarajan N. Pop M. (2009). Statistical methods for detecting differentially abundant features in clinical metagenomic samples. *PLoS Comput. Biol.* 5:e1000352. 10.1371/journal.pcbi.1000352 19360128 PMC2661018

[B52] Whitman T. N. Negri A. P. Bourne D. G. Randall C. J. (2020). Settlement of larvae from four families of corals in response to a crustose coralline alga and its biochemical morphogens. *Sci. Rep.* 10:16397. 10.1038/s41598-020-73103-2 33009428 PMC7532448

[B53] Yang F. Mo J. Wei Z. Long L. (2021). Calcified macroalgae and their bacterial community in relation to larval settlement and metamorphosis of reef-building coral Pocillopora damicornis. *FEMS Microbiol. Ecol.* 97:fiaa215. 10.1093/femsec/fiaa215 33059359

[B54] Zheng X. Q. Li Y. C. Liang J. L. Lin R. C. Wang D. R. (2021). Performance of ecological restoration in an impaired coral reef in the Wuzhizhou Island, Sanya, China. *J. Oceanol. Limnol.* 39 135–147. 10.1007/s00343-020-9253-z

[B55] Zozaya-Valdes E. Egan S. Thomas T. (2015). A comprehensive analysis of the microbial communities of healthy and diseased marine macroalgae and the detection of known and potential bacterial pathogens. *Front. Microbiol.* 6:146. 10.3389/fmicb.2015.00146 25759688 PMC4338804

