# Supplementary figures and images for: Bacterial Communities Associated With Healthy and Bleached Crustose Coralline Alga Porolithon onkodes
Source: Front Microbiol. 2021 Jun 9;12:646143. doi: 10.3389/fmicb.2021.646143 (PMC8219876; doi:10.3389/fmicb.2021.646143)

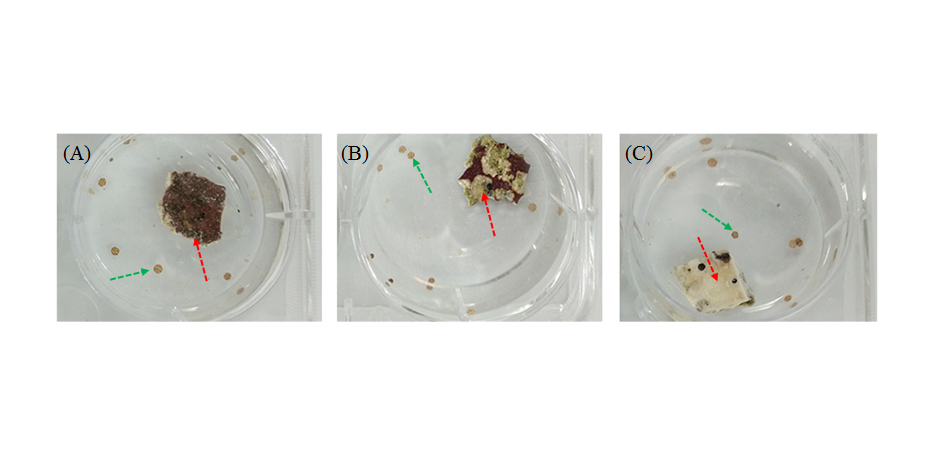

Supplement: Supplementary Figure 1 — Coral larvae exposed to (A) healthy, (B) middle, and (C) bleached P. onkodes. Red arrows represent different health statuses of P. onkodes, while green arrows indicate coral larvae. [file Image_1.TIF]

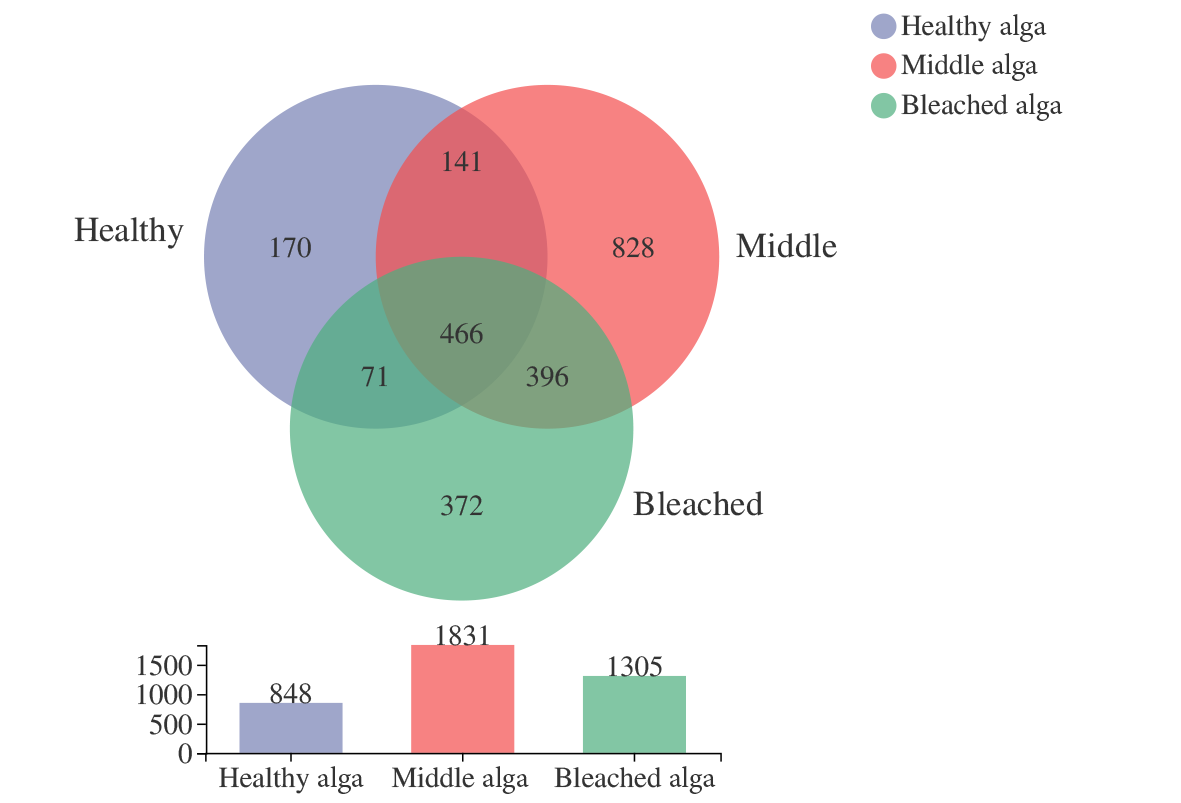

Supplement: Supplementary Figure 2 — Venn diagram showing the number of OTUs in healthy, middle, and bleached P. onkodes based on the 16S RNA gene database at the cut-off level of 97%. [file Image_2.TIF]

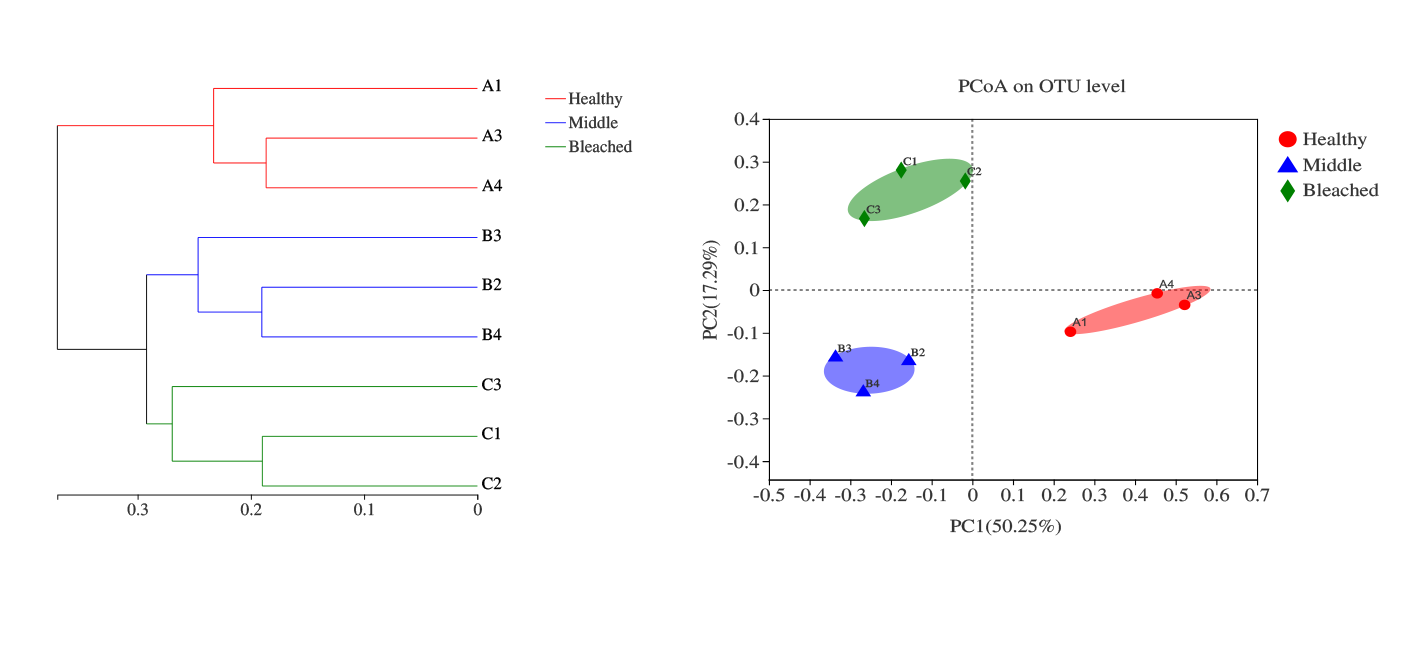

Supplement: Supplementary Figure 3 — The beta diversity of bacterial communities associated with P. onkodes based on a hierarchical cluster tree and principal coordinates analysis (PCoA). The values of axes 1 and 2 represent the percentages that can be explained by the corresponding axis. [file Image_3.TIF]
