# Supplementary material for: Bacterial Communities Associated With Healthy and Bleached Crustose Coralline Alga Porolithon onkodes
Source: Front Microbiol. 2021 Jun 9;12:646143. doi: 10.3389/fmicb.2021.646143 (PMC8219876; doi:10.3389/fmicb.2021.646143)
Supplement: Supplementary Table 1 — Analysis of variance on larval settlement/metamorphosis/swimming rates exposed to different health statuses of P. onkodes. [file Table_1.docx]

Table S1 Statistical analysis of variance on larval settlement/metamorphosis/swimming rates exposed to different health statuses of *Por. onkodes*.

|  | SS | MS | F | Pr(>F) |
| --- | --- | --- | --- | --- |
| Swimming rate | 17.56 | 8.78 | 0.30 | 0.76 |
| Settlement rate | 36.22 | 18.11 | 1.56 | 0.32 |
| Metamorphosis without settlement rate | 4.71 | 2.36 | 0.26 | 0.78 |

SS stands for Sum Sq, MS means Mean Sq.
